# Recombinant C-Terminal Catalytic Domain of Rat L-Gulono Lactone Oxidase Produced in Bacterial Cells Is Enzymatically Active

**DOI:** 10.3390/cimb46080529

**Published:** 2024-08-16

**Authors:** Abdul Aziz M. Gad, Anna Gora-Sochacka, Agnieszka Sirko

**Affiliations:** 1 Institute of Biochemistry and Biophysics, Polish Academy of Sciences, ul. Pawińskiego 5A, 02-106 Warsaw, Poland; annag@ibb.waw.pl (A.G.-S.); asirko@ibb.waw.pl (A.S.); 2 Molecular Biology Department, Biotechnology Research Institute, National Research Centre, Cairo 12622, Egypt

**Keywords:** gulonolactone oxidase, recombinant protein, GULO activity, His-tag, GULO characterization

## Abstract

The L-gulonolactone oxidase enzyme (GULO) catalyzes the last step of L-ascorbic acid (vitamin C) biosynthesis. This enzymatic activity is lost in primates. The full-length rat GULO has been previously produced in plants and demonstrated to be active. In this study, we compared the activity of two variants of GULO produced in *Escheriachia coli* cells, full-length rat GULO (fGULO) and its C-terminal catalytic domain (cGULO). The expression and purification of the recombinant proteins were optimized, and their biological activity was confirmed by two methods, the GULO activity assay in the protein extracts and the ‘in-gel’ staining for GULO activity. Both variants of recombinant GULO were biologically active in both assays. However, cGULO is more promising than fGULO for ascorbic acid production because it is more efficiently produced by bacteria. Furthermore, the optimal activities of the fGULO and cGULO recombinant proteins were observed at pH 7 and 6.5, and at temperatures of 40 and 30 °C, respectively. Kinetic studies revealed that at low substrate concentrations, K_m_ values for fGULO and cGULO were 53.5 ± 5 and 42 ± 6.3 µM, respectively.

## 1. Introduction

L-gulonolactone oxidase (GULO) is required for the final step of L-ascorbic acid biosynthesis. The commercial production of vitamin C has typically been relied on the Reichstein process and two-step methods. However, both methods have a similar drawback: vitamin C cannot be produced directly from D-glucose [1]. Recently, Tian et al. in 2022 [2] introduced an innovative technique for producing vitamin C from D-glucose in *E. coli*. This breakthrough was accomplished through a single-step fermentation process, utilizing the expression of ten genes from *Arabidopsis thaliana*, involved in the vitamin C biosynthesis pathway. Out of the 13 vitamins, ascorbic acid has the highest amount of production in the industry. Approximately 110 kilotons of vitamin C are manufactured annually for industrial purposes, with half used in the pharmaceutical sector, a quarter in the food industry as an antioxidant, and 15% in the beverages industry. Only around 10% of the vitamin is utilized in animal feed purposes. In contrast to other vitamins, the feed sector is the primary application area for vitamins [3]. Although several species are capable of synthesizing vitamin C, this enzymatic activity is lost in humans, guinea pigs, bats, and other primates [4]. The inability of these species to synthesize L-ascorbic acid is due to the absence of a functional *GULO* gene. The majority of lab animals, such as mice and rats, have a functional *GULO* gene. These animals are able to produce ascorbic acid easily when lacking this vitamin in their diet, which undermines their usefulness as models for human vitamin C nutrition [5].

GULO can use as a substrate several hexonic acid lactones with a hydroxyl group on C(2) that matches the configuration of L-gulono-γ-lactone [6]. In this context, GULO catalyzes the conversion of L-gulono-1,4-lactone substrate with oxygen as an electron acceptor to create L-ascorbate and hydrogen peroxide, utilizing L-flavin adenine dinucleotide (FAD) as a cofactor [7,8]. Analysis of the nucleotide sequence of the human genome reveals that the *GULO* gene has accumulated numerous mutations as long as it stopped being active, so that it is now present as a pseudo gene [9,10]. The GULO enzyme belongs to the aldonolactone oxidoreductase family. The enzymes from this family contain two conserved domains: FAD-binding domain (Pfam Id: 01565) at the N-terminus and an arabinono-1,4-lactone oxidase (ALO) catalytic domain (Pfam Id: 04030) at the C-terminus.

The full-length rat GULO has been previously produced in plants and been proven to elevate the level of ascorbic acid and increase plant tolerance to abiotic stresses [11,12]. However, there are no reports on the production of an active recombinant GULO in the bacterial expression system. Recombinant proteins are commonly produced using bacteria as an expression system because of their lower production costs, simple molecular biology, and easy handling. However, the main challenge is the lack of post-translational machinery, leading to the production of inactive proteins caused by incorrect disulfide bond formation. This can result in protein misfolding and aggregation into inclusion bodies [13]. In this study, we focused on the usage of such an expression system for the production of the enzymatically active GULO enzyme. Assuming that the C-terminal cytoplasmic domain of GULO possesses catalytic activity, not only the full-length GULO but also the truncated variant composed of only the C-terminal domain of GULO were produced in *Escherichia coli*. Both proteins were purified based on the presence of C-terminally located His-tag, and their biological activity was demonstrated. Additionally, enzymatic characterization for both variants was carried out as well.

## 2. Materials and Methods

### 2.1. Plasmids, Bacterial Strains, and Chemicals

pET28b(+) expression vector and *Escherichia coli* strains (DH5α, Rosetta(DE3), Rosetta(DE3)pLysS, BL21(DE3)pRARE, HMS174, and C43(DE3)) were obtained from Novagen (Madison, WI, USA). Complete His-tag purification columns were from Roche Life Science Products. All the restriction enzymes, DNA and protein markers, PCR purification kit, and plasmid miniprep kit were obtained from Thermo-Scientific Co., Waltham, MA, USA. The ascorbic acid kit was purchased from Abcam Co., Cambridge, UK. Monoclonal anti-polyhistidine antibody, L-gulonolactone, flavin adenine dinucleotide, reduced glutathione, oxidized glutathione, Triton X-100, phenazine methosulfate, and nitro blue tetrazolium were purchased from Sigma-Aldrich Co., St. Louis, MO, USA. This study used commercially available materials of analytical grade.

### 2.2. Cloning of GULO-Encoding Fragments into Bacterial Expression Plasmids

**Figure 1:**
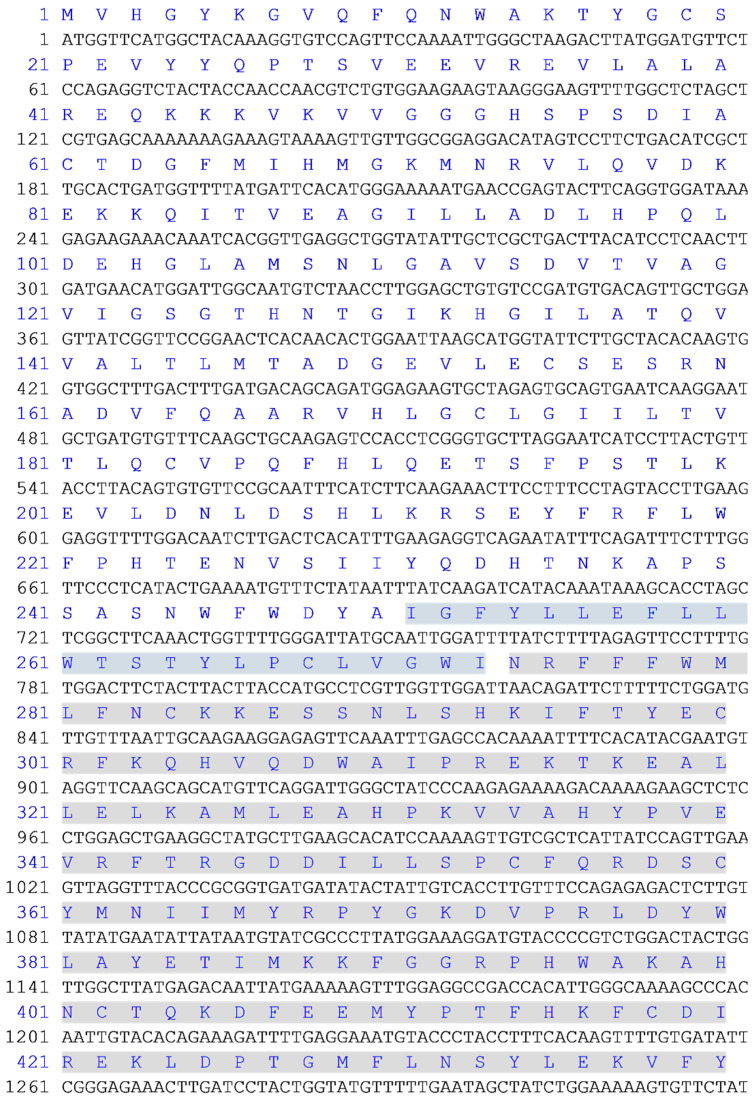
**Nucleotide sequence encoding the GULO protein used in this study**. The deduced protein sequence is indicated above the nucleotide sequence. The non-cytoplasmic domain (amino acids 1–250) is not highlighted, the trans-membrane domain (amino acids 251–273) is highlighted in blue, and the cytoplasmic domain (amino acids 274–440) is highlighted in gray. The domains were identified in recombinant rat GULO by a BLAST search.

The sequence encoding rat full-length GULO (UniProt ID: P10867.3, 1-440 aa.; Figure 1) was synthesized and cloned into the pUC57 vector by GenScript Company, Piscataway, NJ, USA. For further cloning purposes, GULO cDNA were amplified (Kapa HIFi hotstart DNA polymerase, Roche, Basel, Switzerland) using the 5′-TGATCCATGGTTCATGGCTACAAAGG-3′ and 5′-AGCACCATGGATAACAGATTCTTTTTCTGGA-3′ oligonucleotides as forward primers for fGULO and cGULO, respectively, and the 5′-TATTCTCGAGATAGAACACTTTTTCCAG-3′ oligonucleotide as a reverse primer for both products. Following restriction digestion with NcoI and XhoI, the PCR products were ligated into pET28b in frame with the sequence encoding 6×-His tag, resulting in pET28b-fGULO and pET28b-cGULO plasmids.

### 2.3. Production of Recombinant fGULO-His and cGULO-His, and Testing Solubility of Recombinant Proteins

The plasmids were transformed into several different *E. coli* strains to optimize fGULO and cGULO production. Finally, BL21(DE3)pRARE and Rosetta(DE3)pLysS were chosen for fGULO and cGULO production, respectively. For fGULO, the bacteria were cultured in Terrific Broth (TB) with 50 μg/mL kanamycin to OD_600_ = 1, induced with 1.0 mM isopropyl-ß-D-1-thiogalactopyranoside (IPTG), and incubated overnight at 16 °C and 25 °C. For cGULO, bacteria were grown in lysogeny broth (LB) with 50 μg/mL kanamycin to OD_600_ = 0.6 and then IPTG was added to a final concentration of 0.5 mM, and the mixture was incubated at 37 °C for 3 h. Bacterial pellets were resuspended in phosphate-buffered saline (PBS) containing 0.1% Triton X-100 and sonicated. After centrifugation, aliquots of the supernatants and pellets were analyzed using SDS-PAGE.

### 2.4. Protein Purification under Denaturing Conditions

Bacterial cultures (100 mL) were centrifuged, resuspended in 10 mL lysis/equilibration buffer (0.1 M sodium phosphate, (pH 8.0), 0.1% Triton X-100, and 8 M urea), and sonicated using a Branson SX150 sonifier (BRANSON Ultrasonics Corporation, Danbury, CT, USA). The cell lysate was then centrifuged, and the supernatant was applied to a poly-histidine affinity tag column previously equilibrated with the same lysis buffer. There were 10 volumes of wash buffer (0.1 M sodium phosphate (pH 6.5), 0.1% Triton X-100, and 8 M urea). Elution was then performed with elution buffer (0.1 M sodium phosphate (pH 5.5), 0.1% Triton X-100, 500 mM imidazole, and 8 M urea). SDS-PAGE was used to investigate the eluted fractions. The fractions containing the maximum concentration of purified GULO-His recombinant proteins were pooled, and their buffer was exchanged in five consecutive dialysis steps in dialysis buffer (1× PBS (pH 8.0), 2 mM reduced glutathione, 1 mM oxidised glutathione, 2 mM EDTA, 10 µM FAD, and 0.1% Triton X-100) with a gradual decrease in urea concentration (8 M, 6 M, 4 M, 2 M, 1 M, and 0 M). The protein content was determined by Bradford’s method, using BSA as a standard [14].

### 2.5. Western Blot Analysis

Approximately 30 µg of recombinant protein was fractionated by SDS-PAGE and transferred to a nitrocellulose membrane using standard transblotting buffer in an electrophoretic transfer cell (Bio-Rad, Hercules, CA, USA). Following visualization of the transferred proteins with 0.02% Ponceau-S, the membranes were blocked with 5% (*w*/*v*) non-fat dry milk in PBS (pH 7.4) for 1 h and incubated with mouse anti-polyhistidine monoclonal antibody (1:6000). Immunodetection was performed with secondary goat anti-mouse IgG conjugated to alkaline phosphatase (1:10,000, 1 h) and 1-step NBT/BCIP (Thermo Fisher Scientific, Waltham, MA, USA).

### 2.6. GULO Activity Assay

The activity of GULO was determined using a technique adapted from Hasan et al. in 2004 [4] with a few adjustments. The reaction solution (1.0 mL) included a potassium phosphate buffer (50 mM, pH 7.0), 50 mM sodium citrate, 1 mM dithiothreitol, 10 µM FAD, and the enzyme. The reaction began with the addition of 2.5 mM L-gulono-γ-lactone as the substrate and was carried out under aerobic conditions at 37 °C, with vigorous shaking for 15 min, and then halted by the addition of trichloroacetic acid to a final concentration of 5%. Following this, the mixture was centrifuged at 15,000× *g* for 15 min at 4 °C. Ascorbic acid produced in the supernatants was quantified by a colorimetric assay using ascorbic acid assay kit (Abcam Co., Cat. No. AB65346). During the incubation, L-ascorbic acid underwent autoxidation, so the measured value was adjusted to account for this autoxidation that occurred when L-ascorbic acid was added to the reaction without a substrate. A single unit of the enzyme is considered to be the amount that can catalyze the production of 1 nmol of ascorbic acid per minute under the conditions of the assay. The assays were performed in triplicate.

### 2.7. Polyacrylamide Gel Electrophoresis and Staining Gels for GULO Activity

Polyacrylamide gel electrophoresis (8%) was performed following the protocol of Lee et al. in 1999 [15]. To this end, Triton X-100 (0.1%) was incorporated into the gel and the electrophoresis buffer. The proteins were then stained with Coomassie blue. The activity of GULO was marked using the Nishikimi (1976) [16] technique, with slight adjustments. The gels were then incubated in a solution that included 2.5 mM L-gulono-lactone, 0.33 mM phenazine methosulfate, 0.12 mM nitro blue tetrazolium, 1 mM EDTA, 10 µM FAD, and potassium phosphate buffer (50 mM, pH 7.5). The incubation was conducted in the dark at room temperature until the bands clearly appeared, and the reaction was halted by immersing the gel in 7% acetic acid.

### 2.8. Enzymatic Characterization of Recombinant GULO

GULO optimum pH was examined using the following buffers at 50 mM concentration under standard assay conditions: glycine (pH 2.5 to 3.5), sodium acetate (pH 4.5 to 5.5), potassium phosphate (pH 6.5 to 7.5), and Tris-HCl (pH 8.5 to 9.5). GULO pH stability was tested by overnight incubation of the enzyme at 37 °C in the above-mentioned buffers. After incubation, the residual activity was assessed. Thermostability and optimum temperature were determined by measuring the residual activity under standard assay conditions. To study thermostability, the enzyme was left for 30 min at temperatures ranging from 20 to 80 °C, then cooled in an ice-water bath, and finally assayed immediately. Optimum temperature was determined by carrying out the standard GULO assay at a defined temperature for 5 min. The K_m_ for recombinant GULOs was calculated by using a Lineweaver–Burk plot [17].

### 2.9. Statistical Analysis

The data are presented as mean values from (3–5) independent experiments with standard deviation (±SD) indicated. Data analysis was performed using Origin 8.0.

## 3. Results and Discussion

This study reports on production of two variants of His-tagged recombinant rat GULO in *E. coli* cells, full-length (fGULO containing 440 amino acids), and the C-terminal cytoplasmic domain (cGULO containing 167 amino acids due to the deletion of 273 N-terminal amino acids). The low-level expression of *fGULO-His* was successfully augmented by increasing the biomass density (to OD_600_ = 1) and using the low cultivation temperature. Such conditions allow for a slower production of correctly folded protein and a reduction of aggregation [12]. We selected the highest producer, which was in this case BL21(DE3)pRARE [18,19]. Using Terrific Broth (TB) instead of lysogeny broth (LB) resulted in attaining the required cell density for the extended induction time, as previously documented [19,20]. For fGULO, a protein band of expected size, migrating slightly lower than the 50 kDa protein marker, was detected. The expression level of the recombinant cGULO protein was similar in different hosts, and the protein size was in agreement with the expected size of about 20 kDa. The molecular weights of GULO extracted from various sources have been reported to range from 50 kDa for GULO purified from chicken kidney microsomes [6] to 70 kDa for recombinant GULO purified from *Mucuna sempervirens* nectar [21]. Aboobucker et al. in 2018 [22] identified a molecular mass of 65 kDa for recombinant GULO extracted from *Arabidopsis thaliana* (AtGULO5). The variations in molecular weights were due to various modifications in the polypeptide chain after translation, including the number and the glycan chains composition [23].

The fGULO and cGULO had different isoelectric points (pI) and net charges (Figure S1); however, this did not affect their solubility in bacterial cells. Both recombinant proteins were present almost exclusively in the insoluble fraction, and only small amount was recovered from the soluble fraction. In such cases, the common practice is to solubilize the protein using a chaotropic agent, such as urea, which successfully enhances protein solubility [24]. Therefore, the purification procedures were carried out under denaturing conditions (in the presence of 8 M urea in lysis, washing, and elution buffers) to allow for solubilization of the recombinant proteins, following elution with 0.5 M imidazole. To renature the recombinant proteins, we carried out successive dialysis steps of the purified fractions in dialysis buffer, as previously described in Section 2, with a gradual decrease in urea concentration (8 M, 6 M, 4 M, 2 M, 1 M, and 0 M). The first two dialysis steps were carried out using a dialysis bag of molecular weight cutoff of 10 kDa with gentle agitation for 4 h at 16 °C, and the last three steps were performed using a PD-10 (Sephadex G-25, (Sigma-Aldrich, St. Louise, MO, USA)) column according to the manufacturer’s instructions to avoid protein aggregation that started to appear within the dialysis bag at the stage of changing urea concentration from 4 M to 2 M. The dialyzed fractions from PD-10 column were collected, concentrated, and stored as aliquots at −20 °C for further analyses.

**Figure 2:**
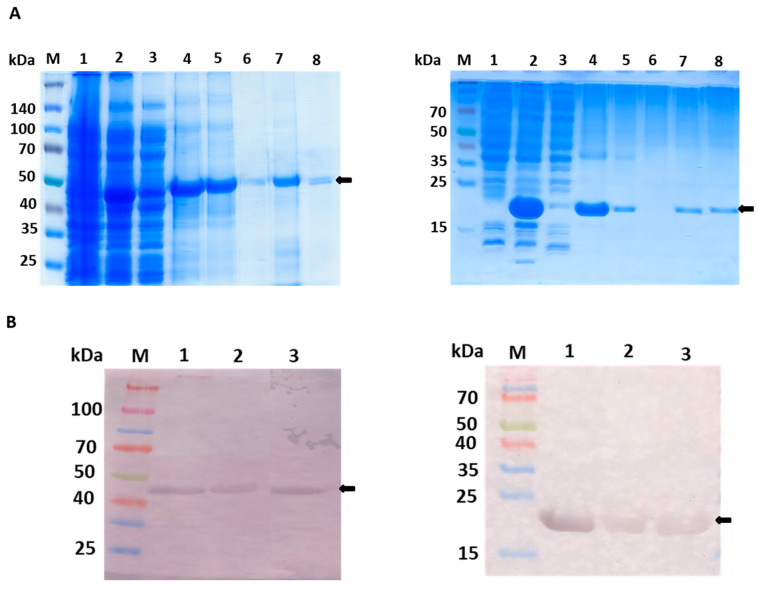
**Purification steps of fGULO-His and cGULO-His using SDS-PAGE** (**A**) **and Western blot with anti-His antibody** (**B**)**.** (**A_left_**) 10% SDS-PAGE of elution fractions from the poly-histidine affinity tag column. M, prestained protein marker with size indicated next to the gel; 1, non-induced lysate of *E. coli* BL21 (DE3) pRARE transformed with pET28b-fGULO; 2, induced lysate; 3, soluble fraction of lysate; 4, insoluble fraction of lysate; 5, flow through; 6, wash; 7, an aliquot of the pooled eluted fractions; 8, an aliquot of the pooled renatured eluted fractions. (**A_right_**) The 15% SDS-PAGE of elution fractions from the poly-histidine affinity tag column. M, prestained protein marker with size indicated next to the gel; 1, non-induced lysate of *E. coli* Rosetta (DE3) pLysS transformed with pET28b-cGULO; 2, induced lysate; 3, soluble fraction of lysate; 4, insoluble fraction of lysate; 5, flow through; 6, wash; 7, an aliquot of the pooled eluted fractions; 8, an aliquot of the pooled renatured eluted fractions. (**B_left_**) M, prestained protein marker; 1, fGULO crude extract; 2, affinity purified fGULO; 3, renatured affinity purified fGULO. (**B_right_**) M, prestained protein marker; 1, cGULO crude extract; 2, affinity purified cGULO; 3, renatured affinity purified cGULO. The arrows indicate the presumed fGULO and cGULO.

**Figure 3:**
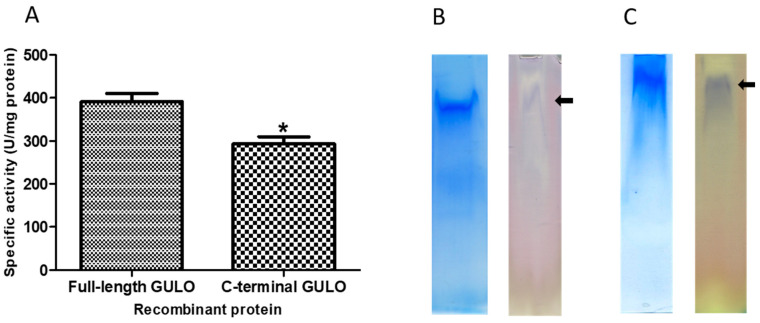
**Verification of enzymatic activity of fGULO-His and cGULO-His recombinant proteins.** (**A**) The GULO activity assays in the protein extracts are presented as means with standard deviation (SD) indicated. One unit of GULO activity is defined as the amount of enzyme capable of production of one nanomole of ascorbic acid/minute/mg protein. The asterisk indicates the statistically significant differences in the specific GULO activity from fGULO, * for *p* < 0.01. The ‘in-gel’ GULO activity assay of fGULO-His (**B**) and cGULO-His (**C**). The respective gel slices, containing the partially purified recombinant fGULO or cGULO proteins, were either stained with Coomassie blue or assayed ‘in-gel’ for GULO activity. The arrows indicate the position of bands visualized by staining for GULO activity.

The amounts of recombinant fGULO and cGULO proteins obtained after purification steps were monitored (Figure 2). Enzymatic activity for recombinant proteins was verified either directly in protein extracts or ‘in-gel’, after protein separation in a native PAGE (Figure 3). The GULO proteins in insoluble fractions of bacterial extracts were first solubilized, purified, renatured, and assayed for GULO enzymatic activity. The protein extract from non-induced bacterial strains was used as a negative control. Both GULO variants were enzymatically active; however, the specific enzymatic activity of cGULO, consisting of only the 167 C-terminal amino acid was significantly lower (about 75%) than that of fGULO, consisting of 440 amino acids. It could be attributed to impaired protein folding, to a decoupling the FAD-binding domain from the catalytic domain, or to the higher proteolytic degradation of the truncated variant as opposed to the full-length variant in bacterial cells [25]. The native PAGE of the renatured affinity purified recombinant fGULO and of cGULO revealed only one protein band in each preparation (Figure 3). Similarly, in the preparations of fGULO and cGULO, a single corresponding band was also detected using the ‘in-gel’ assays for GULO activity. The GULO enzymatic activity resulted in the formation of insoluble blue purple formazan dye (dark band) due to the reduction of nitro blue tetrazolium in the presence of substrate and phenazine methosulphate [6].

The GULO enzyme, which belongs to ascorbate-synthesizing (aldonolactone oxidoreuctases) family, includes two conserved domains: the N-terminal FAD-binding region and the C-terminal HWXK motif, which is specific for D-arabinono-1,4-lactone oxidase (ALO) involved in the final step of D-ascorbic acid biosynthesis. The *Arabidopsis* GULO4 lost GULO activity due to the absence of the ALO domain [26]. The mutations in HWXK motif led to GULO activity loss, as the positions His-447 and Trp-448, which are part of a C-terminal motif, are essential for FMN formation [27,28]. The conserved Lys residue in this motif (Lys-450) is not needed for cofactor binding, yet its substitution with Gly makes the protein inactive. This suggests that the absence of activity in these mutations backs the theory that the conserved HWXK motif is vital for creating the active site of the enzyme. In flavoprotein aldonolactone oxidoreductases, a C-terminal histidine residue is covalently bonded to the FAD cofactor, while an N-terminal histidine residue facilitates this bond. This covalent bond enhances the enzyme’s ability to carry out redox reactions, increase cofactor binding, improve protein stability, and prevent flavin modification. The significant increase in thermal stability with excess FAD suggests that the cofactor protects the enzyme from irreversible denaturation or aggregation [29]. Mutation in the FAD-binding region could contribute to the loss of GULO activity in the bat *Pteropus vampyrus* [30]. It could also explain the failure of Biyani and Madhubala (2011) [31] to detect ALO activity in *Leishmania donovani* in the absence of FAD. Rat GULO full-length has been previously produced in transgenic plants and proven to be active in plants such as tobacco and lettuce [11], potato [12], and *Arabidopsis* [22].

**Figure 4:**
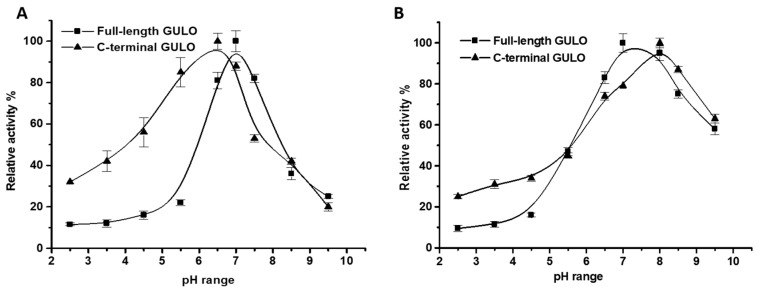
**Effect of pH on the activity of fGULO-His and cGULO-His recombinant proteins.** pH activity profile (**A**) and pH stability profile (**B**). Each data point is the mean of three individual experiments, and the error bars indicate standard deviations.

The impact of pH on recombinant GULO was examined from pH 2.5 to 9.5 at 37 °C. The enzymatic activity of GULO increased steadily in the acidic range of pH 3.0 to 6.0 but decreased gradually in the alkaline range of pH 7.5 to 9.5. Optimal pH values were 7 for recombinant fGULO and 6.5 for cGULO (Figure 4A), consistent with earlier findings by Eliceiri et al. (1969) [32] for rat liver GULO (pH range 6.5–8.3) and by Okamura (2001) [33] for GULO from *Grifola frondosa* (optimal pH 7). GULO activity decreases quickly at higher pH values due to the hydrolysis of lactone substrate. Above pH 8.3, the lactone ring of L-gulonolactone opens rapidly, and at pH 9.0, it is almost completely hydrolyzed nonenzymatically to L-gulonate after 15 min at 37 °C [32]. pH stability of GULO was tested by incubating the enzyme overnight at 37 °C at different pH values ranging from 2.5 to 9.5. The enzyme remained stable within a pH range of 6.5 to 8.5, with fGULO and cGULO variants keeping approximately 75 and 87% of their original activity at pH 8.5, respectively (Figure 4B). It is clear from Figure 4A,B that cGULO is more resistant to acidic pH than fGULO. It is worth noting that pH significantly impacts the ionization of amino acids in an enzyme, leading to decreased stability at highly acidic pH levels due to instability in the enzyme’s binding site [23].

**Figure 5:**
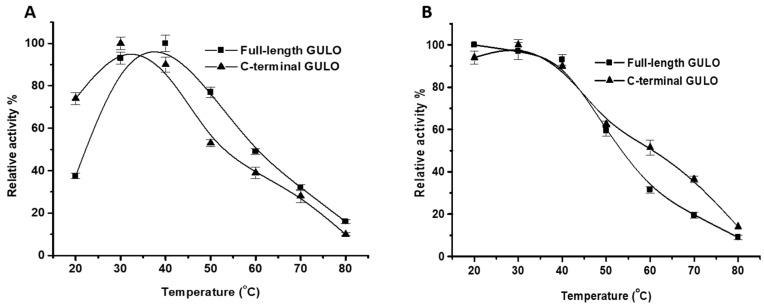
**Effect of temperature on the activity of fGULO-His and cGULO-His recombinant proteins.** Optimum temperature profile (**A**) and thermal stability profile (**B**). Each data point is the mean of three individual experiments, and the error bars indicate standard deviations.

**Figure 6:**
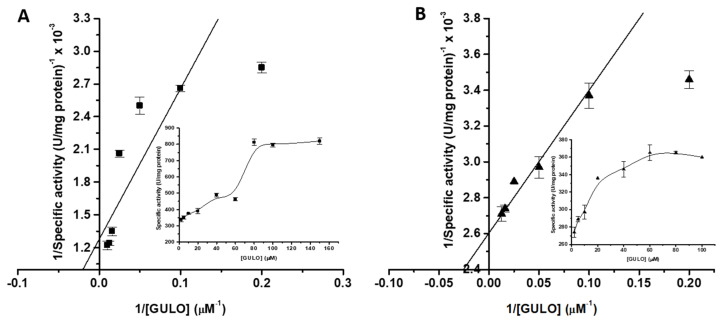
**Enzyme kinetics of the recombinant fGULO-His** (**A**) **and** **cGULO-His** (**B**) **recombinant proteins.** Michaelis–Menten and double-reciprocal Lineweaver–Burke plots are shown. All measurements were made in duplicate in three independent experiments and represented as means with standard deviation.

The activity of recombinant GULO was evaluated at various temperatures from 20 °C to 80 °C to find the optimum temperature. GULO showed the highest activity between 30 and 40 °C. Recombinant fGULO showed maximum activity at 40 °C, while cGULO exhibited its maximum activity at 30 °C; however, at 35 °C, cGULO retained approximately 95% of its original activity (Figure 5A). Our findings are slightly lower than the optimum temperature (45 °C) reported for *Grifola frondosa* GULO [33]. With the rise in temperature, there was a slow decline in the enzyme’s relative activity. At 50 °C, the activity levels were 77 and 53%, while at 80 °C, the enzyme retained just 26 and 10% of its activity for both fGULO and cGULO, respectively. Concerning GULO thermal stability, at 40 °C, both recombinant proteins exhibited high thermal stability with around 90% residual activity. Surprisingly, above 40 °C, cGULO showed more tolerance to higher temperatures than fGULO, retaining approximately 50% of its initial activity at 60 °C compared to only 30% of fGULO. Beyond this threshold, GULO activity began to decline steadily, with the most significant decrease occurring at 80 °C (Figure 5B). Even when exposed to the highest temperature, the enzyme was not completely inactivated, indicating that it may require even higher temperatures or longer treatment times. In this scenario, it was discovered that rat GULO experienced a 90% reduction in activity following a 10 min incubation at 49 °C [25]. The significant rise in heat resistance with FAD suggests that the cofactor shields the enzyme from irreversible unfolding or aggregation. It is believed that a covalent connection between flavin and protein provides a stabilization similar to that of a disulfide bridge [29]. In terms of the substrate affinity and kinetic parameters, the recombinant enzymes fGULO and cGULO were found to have K_m_ and V_max_ values of 53.5 ± 5 and 42 ± 6.3 µM, 780 ± 45 and 374 ± 20 U/mg protein, respectively (Figure 6A,B). The obtained K_m_ values are significantly higher than chicken kidney microsomes GULO (7 µM) [6] and slightly lower than rat GULO (66 µM) [16]. Nonetheless, certain GULO isozymes with elevated K_m_ values have been identified in goat (0.15 mM), *Grifola frondosa* (24 ± 1 mM), and *Arabidopsis thaliana* (recombinant AtGULO5, 33.8 mM) [22].

## 4. Conclusions and Perspective

So far, it is the first report about heterologous production of enzymatically active C-terminal cytoplasmic fragment of rat GULO. The protein was catalytically active, as demonstrated by its ability to produce ascorbic acid from L-gulonolactone in the presence of FAD (cofactor). The cGULO appears to be a more promising option than fGULO for the synthesis of ascorbic acid as it is produced more efficiently than fGULO in bacteria. The presence of His-tagged GULO variants was confirmed through native-PAGE, while SDS-PAGE approximated the molecular weight of the recombinant proteins as 50 kDa for fGULO and 20 kDa for cGULO. Recombinant GULO variants showed their highest enzymatic activity at pH levels of 7 and 6.5 and temperatures of 40 °C and 30 °C for fGULO and cGULO, respectively. In terms of stability, the enzyme showed optimal stability at pH levels between 6.5 and 8.5, as well as temperatures from 20 to 40 °C. Overall, cGULO was found to be more tolerant to acidic pH, thermally stable, and active at low substrate concentrations than fGULO.

Future studies are planned for the production of active recombinant GULO in fusion with proteins enabling polymerization in both bacterial cells and transgenic plants. In the further perspective, such an approach will permit efficient production of the matrix, which will provide the missing enzymatic activity in human tissues, which could be applied topically to wounded skin. It is anticipated that the local content of vitamin C would be increased by externally provided active recombinant GULO, while the polymerizing protein would be used to replace decayed tissues in the wound site.

## Data Availability

Data will be made available on request.

## Supplementary Materials

The following supporting information can be downloaded at: https://www.mdpi.com/article/10.3390/cimb46080529/s1, Figure S1: Comparison of selected physico-chemical properties of full length GULO and C-terminal part of GULO.
