# Supplementary material for: Recombinant C-Terminal Catalytic Domain of Rat L-Gulono Lactone Oxidase Produced in Bacterial Cells Is Enzymatically Active
Source: Curr Issues Mol Biol. 2024 Aug 16;46(8):8958–68. doi: 10.3390/cimb46080529 (PMC11352566; doi:10.3390/cimb46080529)
Supplement: Supplementary file 1 [file cimb-46-00529-s001.zip › cimb-3092531-supplementary.pdf]

Full-length GULO

Isoelectric Point

Isoelectric point:	pI =	**6.850**
Net charge at pH **7.4**:	z =	**-3.398**

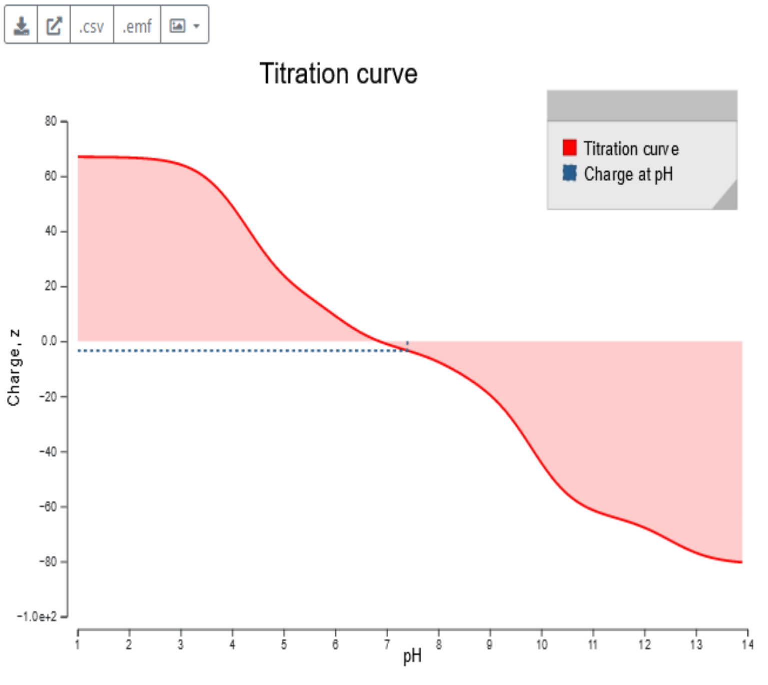

C-terminal part of GULO

Isoelectric Point

Isoelectric point:	pI =	**8.355**
Net charge at pH **7.4**:	z =	**+3.939**

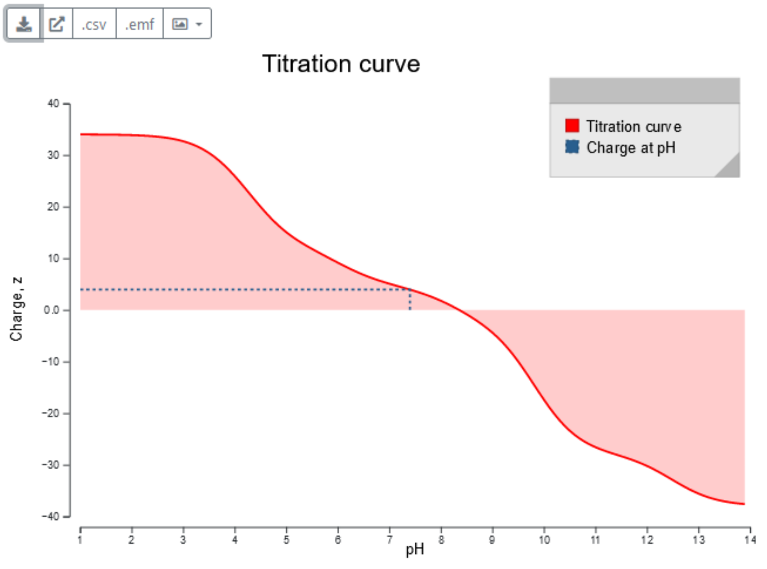

Molecular Mass

Molecular formula:		$C_{931}H_{1389}N_{241}O_{243}S_{13}$
Monoisotopic mass:	m_{mono} =	**20'249.011 Da**
Average mass:	m_{av} =	**20'262.3379 Da**

.csv .emf

Supplementary Figure S1. Comparison of selected physico-chemical properties of full length GULO and C-terminal part of GULO.
